# Exploring nursing students’ learning experiences within tripartite meetings in nursing home clinical placements: a qualitative study using video-stimulated recall interviews

**DOI:** 10.1186/s12912-025-02686-w

**Published:** 2025-01-10

**Authors:** Ingrid Espegren Dalsmo, Kristin Alstveit Laugaland, Stephen Billett, Else Mari Ruberg Ekra

**Affiliations:** 1 Department of Health and Nursing Science, Faculty of Health and Sport Sciences, University of Agder, Grimstad, Norway; 2 SHARE - Centre for Resilience in Healthcare, Faculty of Health Sciences, University of Stavanger, Stavanger, Norway; 3 School of Education and Professional Studies, Griffith University, Brisbane, QLD Australia; 4 School of Nursing, University of Wollongong, Wollongong, Australia

**Keywords:** Assessment, Assessment for learning, Clinical Placement learning, Nursing education, Placement collaboration, Sociocultural learning, Supervision, Qualitative research

## Abstract

**Background:**

Nursing students’ clinical learning is premised on experiences in clinical placements in nurse education, with the processes and outcomes of tripartite meetings among the student, nurse preceptor and teacher being central components. The tripartite meetings form the basis and framework for stakeholders’ dialogue and collaboration and have the central purpose of facilitating student learning and development and assessing the students’ achievement against predetermined learning outcomes for the placement period. Students’ experiences with tripartite meetings seems to be an underexplored field, and therefor this study aimed to explore first-year nursing students’ learning experiences within tripartite clinical placement meetings in nursing homes.

**Design and methods:**

A qualitative explorative and participatory approach was adopted in this study, using the video-stimulated interview method “stimulated recall— dialog and reflection”. This method is based on video recordings with subsequent interviews, where video excerpts were used to support reflection and dialogue. Twenty-one video-stimulated recall interviews were conducted with first-year nursing students (*n* = 7) to explore their learning experiences within the tripartite meetings.

**Findings:**

Data was analysed using reflexive thematic analysis as described by Braun and Clarke. Four themes were identified: (1) the importance of structure and preparedness; (2) supportive relations and dialogue essential for learning; (3) a possibility to create a common learning focus; and (4) assessment needs to be comprehensive and performance focused.

**Conclusions:**

This study highlights that tripartite meetings can be an excellent forum to support the nursing students’ learning process in their clinical placements. Still, consistent and systematic approaches to clinical placement supervision and assessment need to be developed continuously. Therefore, the study’s findings suggest that targeted efforts are warranted to optimise and enhance the learning potential offered in tripartite meetings in clinical education, such as paying a greater attention to the start-up conversation and facilitating comprehensive supervisory and assessment content in the meetings.

**Supplementary Information:**

The online version contains supplementary material available at 10.1186/s12912-025-02686-w.

## Background

Nursing students’ experiences in clinical placements (i.e. learning environments) comprise a significant and essential part of nursing education programmes and are broadly acknowledged as being indispensable elements in the development of nursing competencies [1] and formation of professional identity [2]. In Norway, the bachelor’s program in nursing consists of 50% theory-led studies and 50% clinical placements, with the first-year clinical placement taking place in nursing homes [3]. Supporting nursing students’ learning during clinical placements is usually jointly organised and enabled by educational and healthcare institutions, including the enactment of student supervision and assessment [4].

In Norway, similar to other European countries, the primary clinical placement model is facilitated through a preceptorship model [5, 6]. This model comprises daily supervision of the student by a registered nurse (i.e. a preceptor) alongside monitoring and supervision by a nurse teacher at a higher educational institution [5]. Productive collaborations among these stakeholders are reportedly helpful in supporting and guiding students’ learning and development in their clinical placements [1].

A central component of the preceptorship model is supervision and assessment meetings with the nursing student, nurse preceptor and nurse teacher, i.e., tripartite meetings. The tripartite meetings are usually organised as a start-up conversation, a midterm assessment and a final assessment [6]. These tripartite meetings form the basis and framework for stakeholders’ dialogue and collaboration and have the central purpose of facilitating student learning and development and assessing the students’ achievement against predetermined learning outcomes for the placement period [6, 7].

Research on tripartite meetings has highlighted the importance of participants’ preparation [8], as well as providing students with constructive feedback directed towards enhancing learning and professional development [9]. However, a recent Norwegian observational study, which found that learning opportunities in these tripartite meetings are often suboptimal, called for the need to optimise the learning potential offered in these meetings [6]. Further, different challenges for stakeholders have been identified regarding the assessment of students’ competencies [10, 11]. For nurse preceptors, securing the right balance between patient care and mentoring students is difficult [4]. Also, having to supervision and assess the students simultaneously can affect and challenge the student-preceptor relationship [11]. Nurse preceptors are often not trained to assess student learning and performance, and therefore, preceptors may be uncertain when assessing students’ achievements [9, 12].

On the other hand, nurse teachers are usually geographically removed from assessing student learning in the clinical setting, and instead rely on judgements formed through organised student meetings, written tasks, preceptors’ assessments and students’ self-assessments [12]. For the students, assessment can be intimidating and stressful, and the power relationships between the stakeholders may be unequal, particularly during the midterm and final assessment meetings [13–15]. Yet, research is lacking on how tripartite meetings (including formal and summative assessments) can enhance student learning [6, 9, 15]. Moreover, by better understanding students’ learning experiences, nursing education institutions can address their needs and concerns, thereby fostering enhanced student learning and professional development opportunities. Further, positive nursing home placement experiences may increase nursing students’ interest in the care of older people [16, 17].

Tripartite meetings serve as formal supervision and assessment fora in which nursing students, preceptors and teachers all focus on the students’ learning, and these opportunities can enrich students’ learning experiences in clinical placement. Yet, to date, students’ experiences with tripartite meetings seems to be an underexplored field [14]. Therefore, to address this research knowledge gap, this study aimed to explore first-year nursing students’ learning experiences within tripartite clinical placement meetings in nursing homes.

## Theoretical framework

Theories of sociocultural learning and assessment for learning (AfL) serve as explanatory bases in the appraisal developed and advanced in this paper. Sociocultural theoretical premises are presented as the basis for understanding how individual learning takes place through social interactions in the tripartite meetings. Propositions associated with AfL are explained to understand how assessment practices can support and enhance students’ learning in clinical placements.

### Sociocultural learning theory

Applying a sociocultural perspective in professional education settings suggests that learning arises through (and is shaped by) activities, dialogue and interactions in the social contexts of practice, as elaborated through sociocultural theories of learning [18–20]. These theories propose that learning arises first through social interaction (i.e. inter-psychologically) and second through individuals’ internalisation of social behaviours (i.e. intra-psychologically) [19]. Learners move from potential to actual development within the “zone of proximal development”, while being supported and guided by others through interaction [18, 19], which is sometimes referred to as scaffolding [21].

Central to these processes is the ability to create intersubjectivity (i.e. shared understandings) among interlocutors. That is, by sharing, articulation and discussion, a shared understanding arises between participants with differing levels of experience (e.g. preceptor, teacher and student) [22]. Hence, these conversations and discussions about student performance are important foundations for a novice (i.e. student) to develop understandings and practices from more experienced practitioners (i.e. preceptors and teachers). More generally, participation in practice (as provided by a clinical placement) is therefore the main activity through which effective professional learning can arise situationally [19, 23].

Learning in clinical placements comprises a duality between the experiences afforded and how learners (e.g. nursing students) choose to engage with them [18]. Hence, student-centred approaches to teaching and assessment are promoted in sociocultural learning theory and practice [24, 25]. Here, the required learning is conceptually aligned with patient-centred care, with both approaches being relational, generative and respectful for the lived experience [24]. Consequently, sociocultural theories of learning can help in understanding learning that occurs in clinical placements, including the findings from the investigation reported and discussed here. Whilst this basis for understanding student learning is important, how that learning is supported, enhanced and assessed is critical.

### Assessment for learning

AfL is a well-established concept within higher education pedagogy in Western countries [25]. It signifies a shift in the foundational thinking about assessment by describing the powerful role it plays in shaping how and what students learn. Thus, AfL prioritises the learning function of assessment over its generally more dominant role of grading and certification [25]. Hence, assessment ceases to be solely about evaluating student achievement, but equally about shaping the learning that will arise by stimulating, motivating and challenging learning [12]. On this basis, AfL necessitates feedback to help and guide student learning.

Effective feedback is based on clear, meaningful and timely advice that participants can understand, engage, and act upon [26, 27]. Effective feedback is known to increase student confidence, motivation and self-esteem, and it can also promote positive learning relationships [28]. Acting on feedback requires guidance on how to improve future work, referred to as ‘feed-forward’ (an important aspect of AfL) [25], as ultimately it is the students’ thinking and acting that are central to the learning, performance of work and ongoing development. A positive assessment culture encourages students’ active participation—e.g. self-assessment, where students assess their competencies against a set of criteria outlined for each of the learning outcomes in a course [25, 26]. To optimise conditions for this self-assessment, consistency between assessment tasks, learning activities and objectives (i.e. constructive alignment) is required [29]. Self-assessment and self-reflection can enhance the learning skills that students need for professional competence, thus making them aware of (and more responsible for) their own learning processes [25].

## Design and methods

A qualitative explorative and participatory approach was adopted in this study as a means to elaborate these phenomena. This includes using the video-stimulated interview method “stimulated recall— dialog and reflection” [30, 31], a retrospective think-aloud advanced interview technique designed to enable interviewees to ‘relive’ a situation by presenting it with a stimulus from the original situation—in this case, a video of them acting and interacting [30–32]. It represents a novel approach to explore students’ learning experiences with tripartite meetings in clinical placement.

This study, part of a larger project aiming to improve quality in clinical placement in nursing homes [17], was performed in accordance with the Consolidated Criteria for Reporting Qualitative research (COREQ) [33]. See Appendix A.

### Setting

**Fig. 1:**
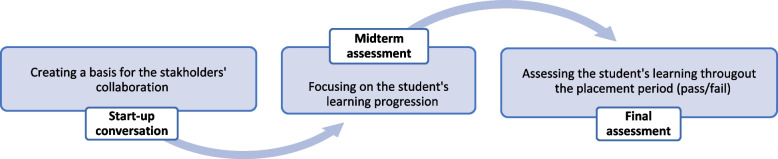
Tripartite meetings in clinical placement

The study was conducted at two Norwegian universities and at five publicly funded nursing homes in three different municipalities associated with the enrolled universities. The nursing home placement involved 8 to 10 weeks of obligatory placement during the nursing students’ first academic year of their bachelor’s degree programme. The preceptorship model included three tripartite meetings during the placement period (see Fig. 1). In this study, the tripartite meetings were held either at the nursing homes or digitally due to COVID-19 pandemic restrictions at the time. The meetings lasted from 16 to 89 min (an average of 45 min). Next, we elaborate on the organisation, purpose and agenda of the tripartite meetings in clinical placement relevant for the setting of this study.

#### Tripartite meetings in clinical placement

The purpose of the start-up conversation, which is the first tripartite meeting, is to create a basis for the stakeholders’ collaboration by addressing roles, responsibilities and mutual expectations [13]. In addition, plans for reaching predetermined learning outcomes are often outlined in these meetings. The second meeting is a midterm assessment, in which the student’s learning progression is the focus of interactions, including making plans for further learning and development during the remaining period. It concludes with a judgement determining whether the students’ competencies are “as expected” [14). The third meeting is the final assessment, held at the end of the placement period. Here, the student’s achievement of the predetermined learning outcomes for the clinical placement is assessed summatively. The student usually begins the meeting with a self-assessment of their performance, followed by assessments by the nurse preceptor and the nurse teacher. A summative assessment form is then completed, usually with a pass or fail result [34]. The formative and summative assessment provided in these meetings is meant to encourage student’s continuous learning process and to provide certification of the student’s achievements [6].

### Participants

Participant recruitment was based on a purposive sampling strategy to generate insights and an in-depth understanding of the target group [35]. The recruitment was conducted stepwise, since nurse teachers and nurse preceptors, in addition to nursing students, had to be recruited for the video recordings of the tripartite meetings. After teachers and preceptors were recruited, first-year students associated with the teachers and preceptors were approached and invited on an ongoing basis. When planning the study, a preliminary estimate was to enrol 6–10 participants due to the comprehensiveness of conducting video-stimulated recall interviews [36]. Seven students (*n* = 7) gave their consent to participate in the study. All participants had Norwegian cultural and linguistic backgrounds. The participants’ ages ranged from 19 to 27, with a median age of 22 years. Five were females, and two were males. Only one of the participants had received other health education, and four had worked in health work. The participants in this study represented a normal cohort in the nursing student population, apart from their homogenous (Norwegian-only) cultural and linguistic background.

### Data collection

Data were collected by the first author from February to June 2021. During this period, the seven participants were interviewed three times each, giving a total of 21 individual video-stimulated recall interviews (*n* = 21). First, the tripartite meetings were video recorded either using a video-camera in the meeting-room (*n* = 8) or by recording the digital meeting (*n* = 13). All stakeholder groups (i.e. nurse preceptor, student nurse and nurse teacher) participated in all three meetings, except for one nurse preceptor who was not present at the start-up conversation. Proceeding each tripartite meeting, the participants and researcher viewed the recordings separately. The participants accessed the recordings through dedicated Microsoft Teams Rooms (version 1.5.00.14473) software. After 2–5 days, the individual stimulated recall interviews were conducted either in person at the nursing homes (*n* = 7) or digitally using the video communications tool Zoom (*n* = 14) [37]. The interviews lasted between 20 and 72 min, with an average of 46 min, and were recorded using a digital recorder or the recording function in Zoom.

**Fig. 2:**
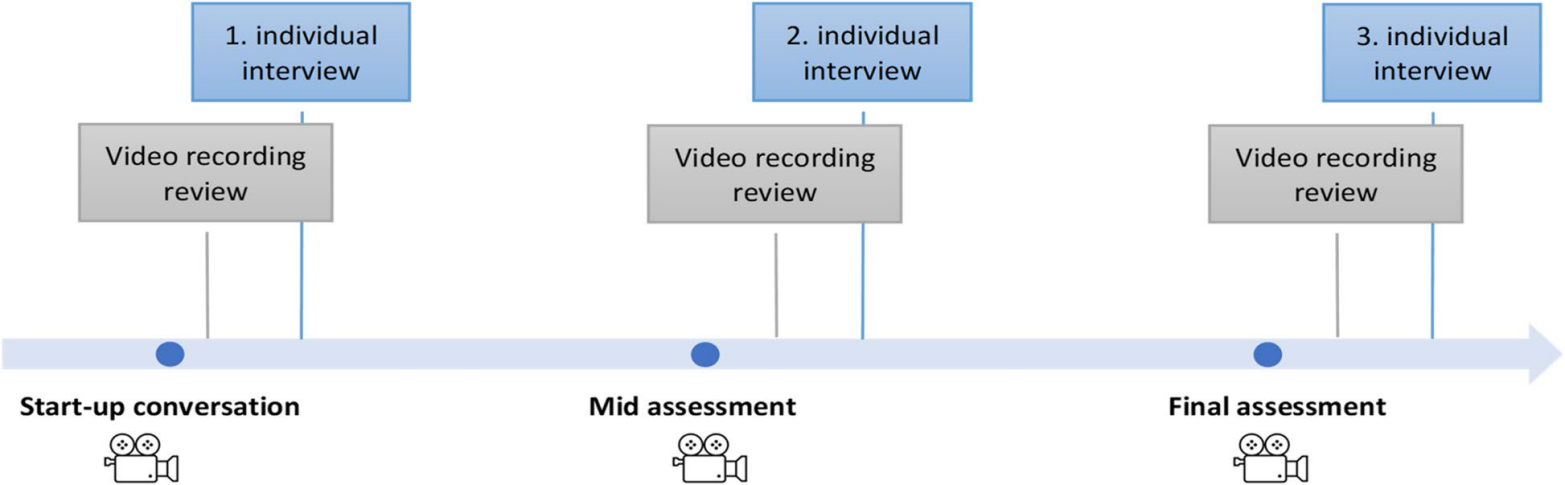
Outline of data generation

Figure 2 depicts an outline of the practical application of the method by showing the tripartite meetings on a timeline with illustrations of the video recording review and individual interviews.

A semi-structured interview guide was developed to facilitate the interviews and enhance consistency and reliability (Appendix B). This guide was created by the research team, drawing on relevant literature and input from experienced researchers in the field. The interview guide was pilot tested, and some changes were made connected to the degree of openness and the possibilities for reflection on the questions asked. For each interview, a complimentary interview guide with possible topics and video extracts, along with time stamps for replay, was prepared. To ensure a person-centred and participatory approach [38], participants were initially invited to share their responses, reflections, and recall of experiences after viewing the recordings, and to discuss any situations they wished to address. During the interviews, short video extracts were replayed on the computer, initiated by both the participants and the interviewer. These extracts were used in different ways. These included at the beginning of the interviews to help participants recall the tripartite meeting, to introduce new themes, and to support or further explore the participants’ experiences. The video acted as a stimulus and resource to bring back the original situation with vividness and accuracy [32]. Moreover, it prompted reflection, dialogue and recall [30], and it was accompanied by questions such as: That is interesting, what was going on there? How did you feel in this situation? Why did you make the choices you made in the situation? Can you tell me more about?

### Ethical considerations

The study was approved by The Norwegian Centre for Research Data (ref. no. 489776) and and by the Etichal Committee at the Faculty of Health and Sports Sciences at the University of Agder. All methods were carried out in accordance with the ethical principles of the World Medical Association Declaration of Helsinki [39]. Informed written consent was obtained from all participants. The students were informed of their right to withdraw from the study at any time and they were made aware that participation or non-participation would not affect any aspects of their placement period or would give advantages or disadvantages during their education programme. The authors were not involved in the clinical placement course and had no prior teacher relationship with the participating students.

Power relationships in the tripartite meetings were discussed in all stages of the study as the students are in a vulnerable situation while being assessed. However, recruitment of the students was relatively easy, implying that the students themselves did not view participating as problematic. The nurse preceptors and teachers who were present during the video recordings of the tripartite meetings also provided informed written consent. Prior to the study, institutional approval was obtained from the two universities and five nursing homes.

### Analysis

The interviews were transcribed verbatim, partly by the first author and a professional transcriber, and made a total of 188 pages of transcriptions. The transcripts from the professional transcriber were quality checked by listening through the interviews while reading the transcribed text. The text was organised using NVivo 12 software [40]. The transcribed interviews were the subject of analysis, not the video of the meetings. Reflexive thematic analysis, as described by Braun and Clarke [41, 42], was used to analyse the data. This is a flexible approach for analysing qualitative data and covers all aspects of the analytic process. The analysis was guided by the research question and followed six phases: (1) familiarising yourself with the data; (2) coding; (3) generating initial themes; (4) developing and reviewing themes; (5) refining, defining and naming themes; and (6) writing up [41, 42]. Even though the interviewers were solely by the first author, all authors participated in the analysis. We strove for a reflexive and thoughtful engagement with the data and the analytic process at all phases [41, 42].

In Phase 1 of the analysis, the first author read through the transcripts to get a general impression of the data. In Phase 2, the transcripts were coded manually by the first author to highlight meaning units relevant to the research aim. The coded data were then merged into potential and recurring themes (Phase 3) addressing the students’ experiences with the tripartite meetings. Similarities, differences and contradictions within the themes were identified, sorted or combined. All authors met to discuss and reach consensus by reviewing, modifying and making final refinements to the themes (Phase 4 and Phase 5). Subsequently, the report was finalised in Phase 6.

## Findings

**Fig. 3:**
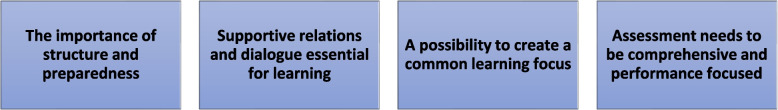
The thematic meaning structure illustrated by the four themes

The analysis identified four themes related to nursing students’ learning experiences within tripartite meetings in nursing home placements: (1) the importance of structure and preparedness; (2) supportive relations and dialogue essential for learning; (3) a possibility to create a common learning focus; and (4) assessment needs to be comprehensive and performance focused. See Fig. 3.

Illustrative quotations from the participants were used to characterise and illustrate each theme. After each quote from the participants, a number and letter were assigned. The number represents a student identifier (student 1 to 7), and the letter stands for which interview the quote was taken from (A = interview after the start-up conversation, B = interview after midterm assessment, and C = interview after final assessment). Quotations from all participants (1–7) and all stages of the interview process (A, B, C) are included in the presentation of the findings.

### The importance of structure and preparedness

During the interviews, the nursing students reported that their learning experience within the tripartite meetings was influenced by their experience of structure of, and preparedness for, the meetings. The participants reported that these two aspects led to predictability, which further affected their feeling of safety in the meetings.

By providing a structure to the clinical placement, most students considered that the tripartite meetings acted as an important link between the university and the nursing home and reminded them about their educational role as students in this setting. One participant said:

*When we’re here at the nursing home*,* it does not feel very much like we’re at school really. So*,* in a way*,* these meetings become a link between the school and here.* (2 A)

Further, having three planned meetings with the preceptor and teacher contributed to this structure and represented a framework for the students’ learning during the clinical placement. The durations and locations of the meetings differed, and the participants reported that these external factors influenced the efficacy of the meetings and consequently their learning experience. The tripartite meetings were usually preplanned in advance, but some were more ad hoc solutions. The meetings were either held in busy staff rooms or in quieter meeting rooms in the nursing homes and varied in duration. Meetings were sometimes brief due to busy shifts, changes in the nurses’ work plans, or the teachers’ tight schedules. Overall, those meetings without haste or disturbances were perceived by the participants as being positive for their learning experience, whereas short or interrupted meetings were perceived negatively. One participant who experienced a short start-up conversation with several interruptions expressed how this influenced the meeting:

*It was very limited what we could talk about. […] I would have liked to have been able to go into that document [learning outcomes] a little more thoroughly. So that the conversation had a little more meaning. […] I would have preferred to sit in a meeting room or group room and have it a bit quiet around us so we could focus a little more. (*7 A)

For the students, predictability was connected to how informed they felt about the meetings. Overall, receiving prior information about the organisation and agenda of the tripartite meetings was reported by the students as being important for their active participation and thus their learning experience. The participants stated that the three stakeholders often came to meetings with different starting points. The teachers and preceptors often had been in similar meetings but not the students. Many of the students felt uncertain and in an unsafe environment when entering meetings on this basis. One of the students explained this uncertainty:

*I think it’s difficult to go to a meeting without knowing what we’re going to talk about*,* and what it’s about. And I do not get to prepare properly either*,* so I think it’s a bit scary really*,* to just go into it like that.* (7B)

Several students wanted the teacher to set a clear agenda at the start of the meetings, and if not implemented, this negatively influenced the students’ learning experience within the meeting. A student who experienced the absence of a framework in the final assessment meeting stated:

*Well*,* it was straight to it*,* without any agenda. I guess meetings usually start with kind of an opening*,* and like “we will go through this and this today” and stuff. I missed that.* (7 C)

To what extent all parties were prepared for the tripartite meetings reportedly influenced the students’ learning experience within the tripartite meetings. Variations were found in how much the students were prepared (self-assessment, review of learning objectives and learning outcomes) and how the students perceived that the preceptor and teacher were prepared. It was perceived to be a positive experience if the student and preceptor prepared for the meetings together. Preparing for the assessment, by going through the assessment form with the nurse preceptor, influenced the students’ feeling of apprehension about going into the midterm and final assessments, as this quote illustrates:

*I wasn’t really that nervous about that meeting. […] [Before the final assessment] we sat down and looked at it [the assessment form] so that we were prepared*. (1 C)

In the collaboration between the three stakeholders, clarification of mutual expectations and responsibilities contributed to creating a structure, which the students valued highly. Most students emphasised the importance of articulating these clarifications from the start of the placement (i.e. during the start-up conversation). In the interview after the start-up conversation, one of the students explained:

*It is important to talk about the framework around the placement. That both the preceptor and student get a clarification of what is to be done and how it should be.* (7 A)

Expectations here included the student’s own responsibility for their learning and the preceptor’s responsibility to facilitate learning in the clinical setting. Altogether, the participants highlighted the teacher’s important role in supervising the students and preceptors in their daily collaboration, as explained by one of the participants:

*The supervision from outside [i.e. nurse teacher] was pretty good. I have noticed that the [learning] curve has gone up after that meeting [midterm assessment].* (4B)

As this quote illustrates, the teacher’s supervision in the meetings influenced the students’ learning at the nursing home.

### Supportive relations and dialogue essential for learning

The participants emphasised relational aspects, such as supportive relations and dialogue, as being important for their learning experiences within the tripartite meetings. The tripartite meetings were experienced as a forum that influenced collaboration among the three stakeholders. Getting to know one another helped to create a safe environment in the meetings, which allowed the students to be more open about their learning needs and learning experiences at the nursing home. Indeed, an open and understanding dialogue in the meetings was important for supporting the student’s learning:

*I think it [the conversation] flowed well and they [the preceptor and teacher] were very supportive. At one time in the meeting*,* I got a little emotional*,* and I was met with a lot of understanding and that was very nice. I think we talked well together.* (5 A)

Several students reported that a dialogue based on mutual respect and getting responses to what mattered most to them led to a feeling of being seen and heard, thus promoting a safe learning environment within the meetings. The students’ linked apprehensions before the tripartite meetings to their relationships with the two other stakeholders, especially the nurse preceptor.

In this study, none of the students knew their assigned preceptor prior to these events, but some knew the teacher from their university courses. The tripartite meetings, especially the start-up conversation, facilitated the chances for the stakeholders to get to know each other. Several of the students stated that if the preceptor and teacher had known about their learning needs, then individual considerations could be made. For example, after watching a video extract from the start-up conversation, one of the participants said that if the teacher and preceptor knew about his [lack of] earlier experience with elderly care, learning focus and learning situations could be customised to his level of readiness:

*If I had worked in a similar place before*,* she [the nurse preceptor] could have focused on other things to help me in my learning process […] So*,* it is good for her [the nurse preceptor] to know that I don’t have any earlier experience with elderly care*,* that I start with blank sheets in a way.* (2 A)

By talking about the students’ personalities connected to their learning needs, considerations could be made in the daily collaboration between the nurse preceptors and the students. For example, one student expressed that it was important for her that the preceptor knew how to handle her shyness:

*I think that [getting to know each other] can have a lot to say*,* because then she knows that I may not be the one… that I’m not so forward in a way. And then she knows that she must push me a little. If not*,* there is a good chance that I do not learn as much.* (1 A)

In turn, getting to know each other also influenced the students’ openness and engagement in the meetings. After the midterm assessment, one student reflected on the emotional dimension of their relationship with the preceptor and teacher:

*I think if you feel safe*,* and I do feel safe with both the teacher and preceptor*,* then you can talk about challenges and difficult things.* (1B)

During the interviews, the participants’ reported that the emotional support that they received from the preceptors and teachers in the tripartite meetings varied, even though they all expressed a need for this support as they were engaging in new and potentially stressful activities. That is, this was the students’ first placement during their nursing education, the nursing homes were a new setting for many, and consequently some overwhelming situations for the students occurred. Those students who got the opportunity in the meetings to talk about topics such as death, making mistakes, insecurity, the demanding student role, challenging patient meetings, and COVID-19-related situations reported this as being important for their learning experience and growth as future nurses. Looking back at all three meetings, one of the students explained:

*I feel that it is valuable to get to know yourself better and how you manage to cope with situations that may suddenly arise. And it’s good that we have these meetings. Then*,* we can talk about heavy stuff*,* or if there is something that you are struggling with or are unsure of.* (3 C)

Overall, the way in which the relationships among the interlocutors allowed healthy interactions to be generated was central for the students’ learning experiences within the tripartite meetings.

### A possibility to create a common learning focus

The students considered that the tripartite meetings enabled a common learning focus between the stakeholders, even though a discrepancy was found in the degree to which this was done. The meetings served as a forum to talk about the learning outcomes and to translate them into the nursing home setting. Most of the students acknowledged the teacher’s role in this translation, and the nurse preceptor’s role was also acknowledged. By knowing the setting, the preceptor could highlight possible learning situations in the nursing home. In the start-up conversations, planning on how the students could reach the learning outcomes (including prioritising by setting short-term and long-term goals) was done to different extents, as shown by these quotes:

*And I think it would have been useful if the preceptor also had been involved in planning. And that “okay*,* during the first 2 weeks we can focus extra on these objectives*,* and the next weeks we will focus on this”.* (7 C)

*It was good to know what I could prioritise until the midterm assessment at least. […] I think it was useful to go through the learning outcomes.* (5 A)

Still, students thought that the many learning outcomes were overwhelming. Therefore, by talking about them in the meetings, the students reportedly developed an understanding and ownership of the outcomes and saw this as fruitful for their learning. One of the universities had a written task in which the students had to formulate what each learning outcome was about, why this was important to learn, and plan on how to reach it. The students conducted this written task before the start-up conversation, and the document’s content was the topic of the meeting. One of the participants recalled this as being helpful for her understanding of the learning outcomes:

*The very first thing we did with writing what*,* why and how*,* I learned a lot from that. If I had only been given a written plan*,* I would probably have just skimmed through it.* (5 C)

The students also referred to the value of talking about other written tasks in the tripartite meetings, as opposed to just the student meetings. In this way, the preceptor also gained insights, which could be a resource for the students, since the writing tasks were closely linked to the clinical setting, as explained here by one of the students:

*The last work requirement is systematic data collection of one of the patients. […] It’s just as well that the preceptor can get an insight to what we’re going to do. So*,* I wish we had talked about that in the meeting*,* because I will need help from the preceptor.* (7 A)

Again, this is an example of the ways in which the tripartite meetings enabled a common learning focus.

### Assessment needs to be comprehensive and performance focused

The nursing students’ learning experiences in the midterm and final assessments varied. Some participants reported that the basis for the assessment was sparse and shallow and clearly missed reasoning supported by examples from the clinical setting. The two universities had different assessment forms, but both had the learning outcomes listed with graded checkboxes and some spaces for free writing. However, how the assessment form was reviewed varied greatly, from superficial and general to more detailed. Most of the students considered that the focus was more on what they had done and less on how nursing care had been performed. In other words, there were few examples and elaborations of the students’ achievements of the learning outcomes (i.e. the students’ nursing competencies) as one of the participants reflected:

*I think it was more sort of listing*,* you have done this and this. […] There wasn’t much time to talk through the different parts [of the assessment form]*,* because there were a lot of* learning *objectives to assess.* (4B)

Even though most of the students received more or less continuous feedback from their preceptor in day-to-day supervision, the feedback they received in the midterm and final assessments was more formal and had clearer links to the learning outcomes. Also, the students reported that the midterm and final assessments served as a catalyst for more collaboration between them and their preceptors by giving an extra reason to talk together before and after the meetings. When asked by the interviewer if the feedback received in the final assessment gave them confidence about achieving the learning objectives, one of the students spontaneously answered: *“Yes absolutely”* (1 C).

Constructive and concrete feedback with a focus on the students’ learning progression was reported as being particularly educational. Those students who received this type of feedback felt they received important confirmation of their development, as these two quotes show:

*Yes*,* it goes up and down how I feel that I manage in clinical placement. So*,* it’s good to get a confirmation that what you’re doing is good.* (5B)

*Getting out of that door with my head raised*,* thinking “yes*,* I’m on the right track”.* (3B)

When their development was highlighted, it became clearer for the students what they mastered, and it showed what they needed to work on further. Still, an over emphasis on further development without first acknowledging the positive steps in the student’s development seemed to reduce the quality of the learning experience and potential to build confidence. One student remarked:

*It is important to get positive feedback as well as constructive. If it had only been a negative focus all the time*,* it might have been quite demotivating.* (2 C)

Comprehensive assessment in the midterm and final assessments included talking about the content in the self-assessment forms that the students had filled out before the meetings. Students reported that the self-assessment of their learning and development of nursing competencies was demanding, but overall, they were positive about their learning experience, as described in the following quote:

*Of course*,* I benefit from communicating my learning process and what I have learned to others. I notice that I learn from it*,* too*,* talking about what I have learned.* (5B)

Practicing assessment and reflection on their learning were considered useful by the participants. One of the forms used the term “to be confident” in connection to the learning outcomes. One of the participants reflected on that term:

*“To be confident” is difficult for me to assess because my feelings are up and down. […] There are some things when I am asked to do it*,* I will do it easily. But does that mean that I’m confident? It’s kind of the definition of what it means that I find a little difficult.* (4 C)

Further, in the interview, this student said that she would have appreciated if there could have been more time and priority set aside to reflect on the self-assessment aspect. In the final assessment, most teachers helped the students reflect further on the remaining education and future work life. In that respect, the students wanted to know what they needed to further develop in the next clinical placement, as shown in the quotes by two other participants:

*We concluded on what I need to work on further*,* and things like that. So*,* that was okay to take with me.* (6 C)

*Yes*,* I think that should be the focus when you are in a learning situation*,* that you should be better tomorrow than you were today. […] I would say that I will be much more confident going into the next placement period than I was going into the first one here.* (2 C)

Overall, participants considered the comprehensive and performance-focused assessment in the midterm and final assessments to be important for their learning experience in the meetings, and thus, their further development as future nurses.

## Discussion

The findings from this study highlight the complex interplay of multiple (i.e., contextual, relational, individual, processual, and competence-related) factors influencing nursing students’ learning experiences within the tripartite meetings. The findings suggest two key aspects essential to enhance the learning potential offered in the tripartite meetings: (1) creating a safe learning environment and (2) facilitating comprehensive supervisory and assessment content. We argue that the theoretical perspectives of sociocultural learning theory and AfL provides a deeper insight into this study’s findings.

### Creating a safe learning environment in the tripartite meetings

The findings from this study indicate how a safe learning environment in the tripartite meetings is influenced by both contextual and relational aspects, and the link to sociocultural learning theories and situated learning is clear [19, 20]. Contextually, the participants’ learning experiences were related to how they were prepared for the tripartite meetings’ setting. For the students, preparedness mattered, as feeling unprepared led to vulnerability and insecurity among students in the tripartite meetings.

Research indicates the importance of general pre-placement orientation before clinical placement [43], and this study also highlights the importance of specific orientation about the tripartite meetings. Although being fully prepared for tripartite meetings is often not possible, knowledge of the different meetings can facilitate a safe environment, thus enabling the students to focus on their learning. Considering the amount of pre-placement orientation needed, digital solutions, in addition to face-to-face meetings, can be useful because they are easily accessible and facilitate individualised and self-paced learning [44].

The meetings created a structure for the placement period and fora for the collaboration between the student, preceptor and teacher, connected to relational factors in sociocultural learning theory [18, 19]. The importance of positive professional relationships in clinical placement has been found in other studies [1, 16]. Several factors influence these relationships throughout the placement period, such as supervisory continuity, communication and interactions [7].

In this study, the participants recognised the tripartite meetings *themselves* as being essential since they affected the students’ and preceptors’ supervisory relationships in daily learning scenarios in the nursing homes. Similar to findings from a previous study [45], the start-up conversation, in particular, was found to be a forum that laid the foundation for this collaboration by clarifying expectations, roles, and responsibilities.

To facilitate a safe learning environment and support students’ learning experiences, our findings highlight the stakeholders’ dialogue in the tripartite meetings in common with other studies [11] and sociocultural learning theory [18, 19]. In a recent study [6], the meetings’ three-part dialogue was found to be hampered by the dominant role of the teacher. However, in the current study, the *nature* of the relationship between the stakeholders was important, not necessarily the degree of participation by each of them.

Indeed, a supportive and caring environment in the meetings was a prerequisite for the students to talk about situations at the nursing homes that emotionally affected them, such as facing sickness, frailty and death. How these processes contributed to the intersubjectivity generated through engagement with more and less experienced practitioners through these meetings was deemed essential knowledge [22]. This emphasises the importance of having a forum to reflect on challenging situations. By acknowledging the experience of the learner as a starting point while fostering students’ self-awareness, students’ individual learning processes can be met [24]. Thus, individual factors accentuate the need for student-centred approaches in tripartite meetings. As for assessment theories, we argue that our findings support the significance of the relational and dialogical perspectives evident in AfL [25].

### Facilitating comprehensive supervisory and assessment content in the tripartite meetings

The goal of tripartite meetings is to prioritise student’s learning through supervision and assessment [9]. In this study, there was a discrepancy in how this was experienced by the participating students and to what degree the meetings were experienced as beneficial for their learning. Connected to the supervisory content, the findings imply that the tripartite meetings were underutilised to encourage and professionally support the students to interpret and critically reflect on clinical situations. This is disturbing, since deep approaches to learning [46] are relevant to nursing students who need deep learning to apply their competencies in different patient situations and contexts.

Clearly, the three stakeholders brought different perspectives and knowledge into supervision and assessment in the tripartite meetings. By learning through interactions with others [18, 19], the preceptor and teacher can be seen as scaffolders for nursing students in their learning processes and development [21]. Aligned to competence-related factors, we argue that the quality of the preceptors’ and teachers’ pedagogic competencies, including supervision and assessment approaches, seem to have a significant impact on student learning. Nurse educators could enhance their teaching quality by integrating insights into curriculum development and incorporating experiential learning techniques. Preceptors, on the other hand, should focus on enhancing mentorship practices and providing more structured support. Students themselves need to participate actively in order to learn from the supervision and assessment received in clinical placement. Therefore, nursing students should be instructed in strategies for managing clinical challenges and optimizing learning opportunities during clinical placements, such as through reflective practices and self-assessment activities.

In both sociocultural learning theories and AfL, learning is seen as an ongoing process [18, 20, 25], implying the importance of formative assessment. In this study, the students expressed the need for formative assessment to support their learning in clinical placement, which was linked to processual factors in the tripartite meetings. The powerful role of formative assessment (e.g. feedback) is also seen in several other studies [11, 14, 27].

In tripartite meetings, formative assessment can be facilitated by focusing on both feedback and feed-forward, with opportunities for reflection between the stakeholders. However, students can become demotivated and misinterpret or misunderstand the meaning of the feedback received. This miscommunication can result in lost opportunities for learning, as well as student dissatisfaction [14]. We agree with Immonen and colleagues [11], who highlight how further development in feedback practices is needed in clinical nursing education.

Interestingly, the study findings reveal that the assessment in the last meeting, being predominantly summative, also had a formative side. The students, having only just finished their first of several placements, valued getting feed-forward also in this meeting to support and facilitate their longitudinal development, in line with the theory of AfL [25]. When it comes to summative assessment, the participants expressed a need for assessment focusing on the *quality* of the nursing care given to the patients, not just on *what* they had done.

Some of the participants considered that the assessment was like ticking off a checklist and was therefore, in terms of comprehensiveness and usefulness for the students’ learning, unsatisfactory. Similarly, in a previous study [13], few examples of assessing professional reasoning along with observable behaviour were found. These findings challenge the credibility of assessment practices in clinical placement. Additionally, Trede and Smith [12] point out that students in practice-based education often underrate their achievements, to avoid being judged as arrogant and overconfident. Uncertainty and hesitation were found when preparing the self-assessment form [9]. Similarly, the participants in the current study reported the self-assessment as being demanding, but they also recognised it as being positive for their learning. At the same time, the students pointed out the importance of reflecting on the self-assessment in the tripartite meetings.

Like other studies [11, 13], we acknowledge that clinical placement assessment is challenging due to the complexity of learning in a situated context, including relational and socioemotional aspects [20, 47]. Adding to this fact is the dual role of the nurse and teacher as both supervisors and assessors, and a blending of formative and summative assessment [12, 14].

Judging the students’ knowledge, skills and attributes against pre-set learning outcomes [29] without weakening the complexity of learning in a clinical setting [12, 48] can be difficult. Considering these challenges, we argue that both assessment *of* and *for* learning is needed, and all assessment stakeholders need to appreciate and engage with their complex coexistence.

### Methodological considerations

The use of the video-stimulated recall interview method, and the fact that each participant was interviewed three times, contributed to a rich dataset, which can be considered a strength of this study. Video-stimulated recall interviews enabled the interviewer and participants to reflect on the research topics and discuss them together. Participation in the three interviews enabled the students to become acquainted with both the interview setting and the interviewer. In addition, interviewing the participants several times enabled possibilities for bringing up earlier topics that needed elaboration, asking follow-up questions and checking our understanding of earlier statements.

We strove to facilitate researcher–participant relations built on trust and reciprocity. This was especially important, since several of the interviews were digital, which could have negatively affected interactions. Still, after nearly 2 years of digital schooling due to the COVID-19 pandemic, the students were used to this form of interaction and reportedly felt comfortable about it. When analysing the data, we saw that the digital interviews provided rich in-depth data, as the interviews that were conducted in person. Sufficient levels of digital expertise are required by both researchers and participants when using digital tools, and this can be challenging.

Data generation was labour-intensive and time-consuming for both the participants and the researchers. This is also stated in other studies that have used recall interview methods [36]. Nevertheless, feedback indicated that the students experienced more advantages than disadvantages when participating, which was connected to increased levels of reflection and higher role awareness. Due to the time-consuming method, we were restricted to a relatively small sample. However, the in-depth nature of this study and interviewing each participant several times can imply information power [49]. Following the concept of information power [49], the adequacy of the sample size was continuously evaluated during the data generation to provide depth of understanding of the phenomena being studied. Information power was ensured by including participants who had thorough experience with tripartite meetings, strong dialogue and reflection-based interviews, and performance of appropriate thematic analysis [49]. The purposive sampling strategy may have contributed to including only engaged students, which could have limited the findings’ relevance [48]. Still, participant variation was observed, including the level of engagement and nursing competencies.

We had to be mindful of several ethical considerations to address participants’ vulnerability while being video recorded in assessment situations. Thus, detailed information regarding voluntary participation and confidentiality was given to the participants before and again at the onset of each video recording. By video recording the meetings, the researchers did not need to be in the room in person, aiming to reduce the participants’ stress levels. Educational research can be challenging when the researchers are teachers and participants are students, as there are power differences at play [50]. Aiming to reduce this imbalance, the authors were not involved in the clinical placement course and had no prior teaching relationship with the participating students.

## Conclusions and implications

This study contributes to the existing body of knowledge on clinical placement supervision and assessment, by highlighting that tripartite meetings can be an excellent forum to support the nursing students’ learning process in their clinical placements. Still, supported by current literature, the findings imply that consistent and systematic approaches to clinical placement supervision and assessment need to be developed continuously. Therefore, several implications for how to utilise the learning potential of the tripartite meetings for both nurse education and the clinical setting can be identified in this study.

The importance and potential of the start-up conversation, in particular, is evident from the findings, suggesting that greater attention should be paid to this meeting. In addition, pre-placement orientation can assist students’ learning in clinical placement by reducing stress and uncertainty. Moreover, collaborative practices with student-centred approaches, focusing on dialogue and emotional support, need to be pursued to foster student learning in all tripartite meetings.

We argue that all clinical placement assessments should focus on feedback and feed-forward to enhance student learning and professional development processes. The assessment of students’ competencies needs to shed light on the quality of the students’ nursing care with concrete clinical examples. To enable this, supervision and assessment competencies for all stakeholders are needed. Therefore, targeted efforts are warranted to enhance nurse preceptors’ pedagogical and supervisory competence, such as formal education and training. Assessment forms and their characteristics can either promote or hinder formative and student-centred assessment, challenging nursing education to evaluate the quality of the assessment forms used in clinical placement.

Hopefully, the knowledge gained from this study can assist in challenging the perception of and approach to supervision and assessment in tripartite meetings in clinical placement. There remains a gap in the knowledge on the shared experiences of nursing students, nurse preceptors and nurse teachers during tripartite meetings. Further investigation into the interactions within these meetings, such as through conversation analysis, would be valuable. This approach could provide deeper insights into the collaborative dynamics of stakeholders in supervision and assessment practices.

## Supplementary Information

Supplementary Material 1. A: Consolidated Criteria for Reporting Qualitative Research (COREQ).

Supplementary Material 2. B: Interview guide.

## Data Availability

The datasets analysed during the current study are available from the corresponding author on reasonable request.

## Abbreviation

AfL: Assessment for Learning
