# Supplementary material for: Exploring nursing students’ learning experiences within tripartite meetings in nursing home clinical placements: a qualitative study using video-stimulated recall interviews
Source: BMC Nurs. 2025 Jan 10;24:40. doi: 10.1186/s12912-025-02686-w (PMC11724606; doi:10.1186/s12912-025-02686-w)
Supplement: Supplementary file 2 — Supplementary Material 2. B: Interview guide. [file 12912_2025_2686_MOESM2_ESM.docx]

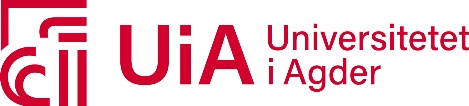
**“Learning in clinical practice**

**- addressing the student nurse perspective”**

**Interview guide: video stimulated individual interviews with nursing students**

**Aim:** *To explore first-year nursing students’ learning experiences within tripartite clinical placement meetings in nursing homes.*

**Research questions:** *1) How do first-year nursing students experience learning support and guidance in the tripartite meetings taking place during clinical practice in nursing homes? 2) During clinical practice in nursing homes, how do first-year nursing students experience the assessment conducted in the tripartite meetings?*

**First Interview**: Information about the project and the interview situation. Personal details (age, gender, previous education/work experience, familiarity with the nursing home/nursing home work, familiarity with the teacher).

Any questions before we continue?

**Introduction:** Now you have insight into this tripartite meeting from both the inside and the outside, while I only have insight from the outside. I would now like to reflect with you to gain increased insight and understanding of your experiences from this meeting.

**Tips for formulating interview questions**

**Stop the recording and...**

- Remain silent

- Give a neutral description of something in the recording (e.g., "You say X here")

- Ask a neutral, open-ended question (e.g., "What is happening here?")

- Present an observation (e.g., "You seem to hesitate a bit here...")

- Ask about intentions/goals (e.g., "What did you achieve with X?")

- Ask an evaluative question (e.g., "What do you think about X?")

**Clarify any unclear dialogue**

What was the reason you did/said X?

What did you understand from what X said here?

What do you think X meant when he/she said that?

Was there anything you decided not to say/mention in the conversation?

**Invitation to the participant - the student is encouraged to show the excerpts from the video recording that he/she noticed and wishes to discuss.**

What do you think about this part of the meeting (can you guide me through it)? How did you experience this situation?

What are your reflections on what is happening here?

Did you experience this as a positive/negative part of the meeting, as relevant and/or useless?

Explain why you chose to highlight this part.

**The researcher shows her excerpts from the video.**

You will now see a part of the meeting where I think something interesting happened. What do you think about this part of the meeting? How did you experience this?

**Possible topics to cover during the interview:**

- Preparation and expectations before the meeting
- Content of the meeting (positive/negative, missed/unnecessary?)
- Interaction/dialogue – student as active/passive?
- The usefulness/relevance/role of the meeting for the student's learning and learning process in placement
- The student role
- Motivation for learning and development
- Feelings of safety and support
- Supervision and feedback
- Justification of the assessment
- Assessment collaboration between the preceptor and the teacher
- Self-assessment and documentation of own learning
- Feedforward

**Conclusion**

Is there anything else you would like to share about what we have talked about today?

*Thank you very much for sharing your experiences and insights in this interview.*
